# The New Face of Dynamic Mutation—The CAA [CAG]n CAA CAG Motif as a Mutable Unit in the *TBP* Gene Causative for Spino-Cerebellar Ataxia Type 17

**DOI:** 10.3390/ijms25158190

**Published:** 2024-07-26

**Authors:** Dorota Hoffman-Zacharska, Anna Sulek

**Affiliations:** 1 Department of Medical Genetics, Institute of Mother and Child, 02-106 Warsaw, Poland; 2 Institute of Psychiatry and Neurology, 02-957 Warsaw, Poland; anna.sulek@lazarski.pl; 3 Faculty of Medicine, Lazarski University, 02-662 Warsaw, Poland

**Keywords:** hereditary spinocerebellar ataxia, SCA17, *TBP* gene, TATA-binding protein, dynamic mutation, CAA/CAG repeat expansion

## Abstract

Since 1991, several genetic disorders caused by unstable trinucleotide repeats (TNRs) have been identified, collectively referred to as triplet repeat diseases (TREDs). They share a common mutation mechanism: the expansion of repeats (dynamic mutations) due to the propensity of repeated sequences to form unusual DNA structures during replication. TREDs are characterized as neurodegenerative diseases or complex syndromes with significant neurological components. Spinocerebellar ataxia type 17 (SCA17) falls into the former category and is caused by the expansion of mixed CAA/CAG repeats in the *TBP* gene. To date, a five-unit organization of this region [(CAG)3 (CAA)_3_] [(CAG)n] [CAA CAG CAA] [(CAG)n] [CAA CAG], with expansion in the second [(CAG)n] unit being the most common, has been proposed. In this study, we propose an alternative organization scheme for the repeats. A search of the PubMed database was conducted to identify articles reporting both the number and composition of GAC/CAA repeats in *TBP* alleles. Nineteen reports were selected. The sequences of all identified CAG/CAA repeats in the TBP locus, including 67 cases (probands and b relatives), were analyzed in terms of their repetition structure and stability in inheritance, if possible. Based on the analysis of three units [(CAG)_3_ (CAA)_2_] [CAA (CAG)n CAA CAG] [CAA (CAG)n CAA CAG], the organization of repeats is proposed. Detailed analysis of the CAG/CAA repeat structure, not just the number of repeats, in *TBP*-expanded alleles should be performed, as it may have a prognostic value in the prediction of stability/instability during transmission and the possible anticipation of the disease.

## 1. Introduction

The human genome contains numerous repeated sequences varying in complexity and organization, ranging from tandemly repeated satellites, typically localized in specific chromosomal regions, to interspersed repeats that generally represent inactive transposable elements but also active RNA genes. The simplest and most common form of these sequences is microsatellites, also known as simple/short tandem repeats (STRs) or simple sequence repeats (SSRs). Microsatellites consist of tandemly repeated DNA units with a repeat length of up to six nucleotides. They are highly polymorphic in populations mainly due to their length variability, which reflects changes in the number of repeated units at a *locus*.

Studies have shown that the expansion of repeat sequences of various lengths can be associated not only with human cancers but also with a growing number of hereditary diseases. Since 1991, several genetic disorders caused by this mechanism, known as *dynamic mutation*, have been described [1]. These disorders, referred to as a family of triplet repeat expansion diseases (TREDs), are characterized as neurodegenerative diseases or complex syndromes with a significant neurological component. The first group is caused by the expansion of [CTG]n or [CAG]n repeats in the open reading frame (ORF), and the second by the expansion of [CGG]n or [CCG]n in 5′ or 3′ untranslated regions (UTRs) or [GAA]n or [TTC]n in introns of the respective genes [1,2]. Among the ten possible trinucleotide combinations, only these three have been demonstrated to undergo dynamic mutation, resulting in genetic diseases. Their combinations, as well as a less common one, [GAC]n or [CAG]n, can form stable secondary structures in vitro if they are long enough [3,4,5]. These sequences can adopt several unusual DNA structures, such as hairpins, triplexes, quadruplexes, and slipped structures, whose formation may disrupt DNA metabolism and serve as critical intermediates in dynamic mutation events [4,6]. The importance of such structure formation for repeat tract instability has been confirmed in vivo in *E. coli* and *S. cerevisiae* models [5]. TNRs are characterized by instability during transmission to the next generation as well as in somatic cells, with various DNA processes, including replication, recombination, repair, and transcription, influencing their stability. The precise mechanisms by which these systems interact to produce expansion or contraction remain unresolved, but the basic mechanism accepted for TNRs’ instability is slipped strand mispairing during replication [6]. In this model, the formation of slipped DNA structures is proposed, leading to repeat expansion or deletion depending on strand localization related to the orientation effect of the sequence. However, this simple model does not explain the characteristic features of human TNR instability, such as the polarity of expansion (usually at the 3′ end of the track) and mutational bias towards expansion rather than contraction [3]. Another model for TNR expansion proposes the formation of FEN-1-resistant secondary structures at the 5′ flap end of the Okazaki fragment. The single-stranded DNA 5′-flap end of Okazaki fragments, originating from strand displacement during lagging strand synthesis, can be removed by flap endonuclease 1 (FEN-1). However, if these ends form structures like foldbacks or hairpins, this process is inhibited. It is also inhibited by the complex of the DNA-flap/single-strand binding protein (SSBP), which could be involved in the unfolding of unusual DNA structures [7]. Experimental systems show that mutations in FEN-1 and its *S. cerevisiae* ortholog RAD27 gene enhance microsatellite instability and spontaneous mutations (mainly sequence duplications). Such mutants have increased recombination rates and require a functional double-stranded break (DSB) repair system to survive [8,9]. These data suggest the additional involvement of recombination processes and replication perturbations in TNR instability. Recent studies indicate that expansion could occur by multiple processes and at different stages of germ-line development: in the pre-meiotic stage by replication polymerase slippage and DNA repair during meiosis by DSB repair and post-meiotically by DNA damage repair [10]. Generally, two types of factors may influence trinucleotide instability: trans-acting factors, including those involved in DNA replication and repair (FEN1, Msh2, Msh3, Msh6), and other factors reflecting specific properties of the loci, such as the presence of CpG islands, orientation, proximity to the replication origin, and the number and configuration of the repeats themselves (pure TNRs are more prone to instability during replication) [11].

The aim of this paper is to conduct a comparative analysis of the repeat region of the *TBP* gene (OMIM: 600075), where a dynamic mutation causes spinocerebellar ataxia type 17 (SCA17; OMIM: 607136). SCA17 is classified as a neurodegenerative TRED and is caused by the expansion of mixed CAA/CAG repeats in the gene-encoding TATA-binding protein (TBP). TBP is a general transcription factor and a component of TFIID, which is a transcription complex that regulates the expression of most eukaryotic genes transcribed by RNA polymerase II, as well as polymerase I and III transcription complexes (SL1 and TFIIIB, respectively) [12]. Neurodegenerative TREDs are caused by the expansion of [CAG] repeats in the open reading frames (ORFs) of the respective genes, leading to long polyglutamine (polyQ) tracts in the proteins. These disorders are thus known as polyglutamine (polyQ) diseases. The [CAG]n stretches in TRED genes are typically pure, but there are exceptions. For instance, non-pathogenic alleles of the spinocerebellar ataxia-related genes *ATXN1* (SCA1; OMIM 164400) and *ATXN2* (SCA2; OMIM 183090) have tracts interrupted by 1–3 CAT (His) codons in *ATXN1* and CAA (Gln) codons in *ATXN2* [13,14,15]. The absence of such interruptions in expanded pathogenic variants suggests their role in maintaining regional stability. A similar role is postulated for the penultimate CAA (Gln/Q) interruption at the 3′ end of the [CAG]n tract in the *HTT* gene (HD; OMIM 143100). Even when present in most expanded alleles, this codon can contribute to instability and expansion into the pathogenic range if mutated in alleles of an intermediate [CAG]n length [16].

The polyQ-coding region in the *TBP* (SCA17) gene is more complex, comprising both CAG and CAA Gln codons in normal and expanded alleles. Since Koide et al. described a sporadic case of cerebellar ataxia with pyramidal signs and severe intellectual impairment associated with CAG/CAA expansion in the *TBP* gene in 1999 [17], many SCA17 cases have been reported, some with detailed repeat sequence analysis. A large population study by Gostout et al. in 1993 [18] identified non-pathogenic alleles corresponding to 25–42 glutamine residues, finding 20 different alleles, most encoding 32–39 glutamines. Based on the sequence of 157 independent alleles, a five-unit organization of the *TBP* polyQ coding region, [(CAG)_3_ (CAA)_3_] [(CAG)nI] [CAA CAG CAA] [(CAG)nII] [CAA CAG], with polymorphic blocks of pure (CAG)n repeats as the primary sites of repeat number variation were proposed [18]. Later studies established the SCA17 polyQ pathogenic range as 41 or more repeats, with reduced penetrance in the 41–48 repeat range, which is especially controversial in the 41–44 repeat range, where pathogenic variants in the *STUB1* gene coexist. This novel mechanism was defined as digenic *TBP/STUB1*-related SCA17 [19]. Recently, however, this mechanism has been questioned, and it was shown that intermediate TBP_40–49_ alleles act as disease modifiers of SCA48 (OMIM 618093) caused by *STUB1* mutations rather than a STUB1/TBP digenic model [20]. The fully pathogenic range is set at 49 repeats or greater, with the largest known allele containing 66 repeats [17,18]. In some SCA17 cases, both the repeat number and the sequence of the mutated region have been analyzed [17,18,21,22,23,24,25,26,27,28,29,30,31,32,33,34,35,36,37,38,39]. The available data suggest three possible *TBP* gene mutations: the expansion of the CAG repeats in (CAG)nII, partial deletion of the region resulting in only one unstable (CAG)n tract, and partial duplication of the repeats region resulting in an expanded polyQ domain. Among the published cases, the most common form of mutation is the expansion in the [(CAG)nII] unit. Based on the available data and identified SCA17 cases, we propose a different view of the region, which is considered a three-unit region, [(CAG)_3_ (CAA)_2_] [CAA (CAG)n CAA CAG] [CAA (CAG)n CAA CAG], with different susceptibility to expansions.

## 2. Results

**Figure 1:**
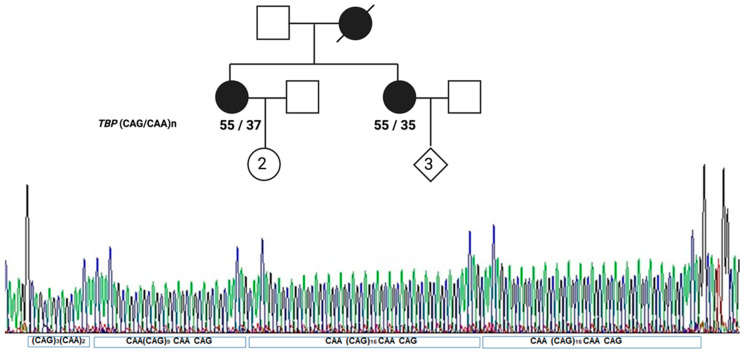
Pedigree and sequence of the CAG>GAA repeat region in the *TBP* gene of a family with two patients with the same number of repeats in the pathogenic allele *n* = 55 and the same repeat configuration. The sequence is given below the chromatogram, where the domain division is also marked according to the scheme we propose. (Created with BioRender.com; accessed on 15 July 2024).

Based on the PubMed search, which was performed to identify reports presenting CAG/CAA repeat region sequences of the *TBP* gene, 19 publications were chosen (listed in Table 1). The analysis of CAG/CAA repeat organization schemes of the SCA17/*TBP* gene was performed for published data as well as for probands and their relatives identified among HD/SCA patient cohorts of Polish origin diagnosed in the Dept. of Genetics Institute of Psychiatry and Neurology [38], unpublished data, Figure 1.

Sequences of all identified CAG/CAA repeats in the *TBP* locus—78 cases—(probands and relatives) were collected and are presented in Table 1. Five different types of repeat configurations were identified, which occurred with different frequencies in the study cohort and were characterized by different inheritance stability. The most common and stable in transmission was configuration (CAG)_3_ (CAA)_3_(CAG)_9_ CAA CAG CAA (CAG)_n_ CAA CAG—65.4% cases, and the second (CAG)_3_(CAA)_3_ (CAG)_n_ CAA CAG—23%. This configuration showed instability in transmission from 1 to 7 (CAG)n repeats. Three other configurations, much rarer, were also identified (CAG)_3_ (CAA)_3_(CAG)_9_ CAA CAG CAA (CAG)_n_ CAA CAG CAA (CAG)_n_ CAA CAG—6.4%, (CAG)_3_ (CAA)_3_ (CAG)_12_ CAA CAA (CAG)_13_ CAA (CAG)_16_ CAA CAG—2.6% and (CAG)_3_ (CAA)_3_ (CAG)_9_ CAA CAG CAA (CAG)_9_ (CAA)_3_ (CAG)_9_ CAA CAG CAA (CAG)_n_ CAA CAG, with 2.6% identified de novo in paternal transmission. Based on the analysis of all those sequences, we propose an alternative model of *TBP* CAG/CAA organization and expansion presented in Table 2.

## 3. Discussion

SCA17 is a typical polyQ neurodegenerative disorder, but on the other hand, unique due to the organization of the polyQ-coding region in the *TBP* gene. The pure polyglutamine track is coded by CAG stretches interrupted by CAA repeats (both coding Gln/Q). As was mentioned, Gostout et al. [18] proposed the organization of these repeats as a five-unit complex [(CAG)_3_ (CAA)_3_] [(CAG)nI] [CAA CAG CAA] [(CAG)nII] [CAA CAG] with two possible configurations according to Gao et al., 2008, as type I and type II, with and without domain III [CAA CAG CAA] [11]. Stretches of type I are more stable somatically and germinally, and expansion usually occurs here in domain IV [CAG]n but more complex rearrangements are also observed, including the partial duplications of domain III—[(CAG)_3_ (CAA)_3_] [(CAG)_9_] [CAA CAG CAA (CAG)_16_ CAA CAG CAA (CAG)_16_] [CAA CAG]—but also more complicated rearrangements including partial duplication [(CAG)3 (CAA)_3_] [(CAG)_9_] [CAA CAG CAA (CAG)_9_] [(CAA)_3_ (CAG)_9_ CAA CAG CAA (CAG)_19_] [CAA CAG] or deletions [(CAG)3 (CAA)3] [(CAG)n] (Table 1). Type II may be a derivative of the original five-component repeat combination, which was created by deletions of domain III [(CAG)_3_ (CAA)_3_] [(CAG)n ] CAA CAG], which destabilizes the sequence of domain II repeats and can easily expand in inheritance parent-offspring.

Here, based on the results from previous studies as well as our own data, a different scheme of the mutable CAG/CAA repeats’ organization in the *TBP* gene is proposed, not as a five-unit [(CAG)_3_ (CAA)_3_] [(CAG)nI] [CAA CAG CAA] [(CAG)nII] [CAA CAG], but as a three-unit tract [(CAG)_3_(CAA)2]_I_ [CAA (CAG)n CAA CAG]_II_ [CAA (CAG)n CAA CAG]_III_ with different mutable components, two of which are identical except for the (CAG)n repeats number (Table 2). The (CAG)n tract in segment II is rather stable, with nine repeats in most cases; the main unstable (CAG)n tract is in the last segment (III). The suggested motifs’ organization indicates three possible ways of repeat region elongation: (a) Expansion of the second (CAG)n domain (Gostout’s unit IV), with its stable transmission in families, as the presence of two repeats of the [CAA (CAG)n CAA CAG] motif stabilizes the whole region and only allows for a slow multistep expansion of the second (CAG)n track. (b) Partial deletion of the region, probably units II and III, followed by the strong expansion in the remaining (CAG)n domain, which is also unstable during transmission, and (c) the partial duplication of the repeats region as a result of the duplication of the unit III or more complicated rearrangements due to the formation of FEN-1 resistant structures during replication.

To date, in the case of a partial duplication, it was not so easy to determine which parts of the region are prone to mutation; the model of repeats organization proposed here may better explain changes involved in the partial deletion or duplication of this region. In the [CAA (CAG)n CAA CAG] segments, even in the normal range, the (CAG)n repeats a number in most cases (>80%) and is long enough to form a hairpin structure (the number of repeats required for expansion/contraction has been estimated as 15/17, respectively) [35]. The loss of one of such motifs destabilizes the repeats’ structure and the remaining (CAG)n track is more prone to expansion by easier formation of the secondary structures during replication and causing the polymerase slippage. Although only two such cases have been published, they clearly show the instability in the transmission leading to the anticipation of the disease inheritance [30,34]. Koide et al. proposed a hypothetical model of de novo expansion in the *TPB* gene by 5′-flap single-strand generation in the Okazaki fragment and formation of the FEN-1 resistant structure (hairpin) which was finally duplicated during replication [17]. The formation of such structures could also be the base of partial deletions when the hairpin at the 5′-flap end of the Okazaki fragment is recognized and processed by FEN-1 nuclease. However, this is probably not the case since FEN-1 is able to process the 5′ flap DNA of CAC/CTG repeats in a length-dependent manner. It has been demonstrated in vitro that human FEN-1 nuclease cuts such molecules containing up to 21 repeats but its activity decreases when the flap has over 11 repeats, and this may be correlated with a growing tendency to form higher structures suppressing FEN-1 activity [40]. A possibly removed hairpin, formed between the whole motif II and part of motif III, would be at the threshold value for such a reaction (about 21 repeats). This might rather suggest a single case of contraction, as a result of the hairpin formation on the lagging strand template, followed in the next generations by (CAG)n expansion in a single and unstable [CAA (CAG)n CAA CAG] motif. Two cases with partial duplication of the repeats’ region, published by Nakamura et al. [22], and the second one presented here also show the stable transmission of such mutated alleles. In both families, the presence of the duplication of the second [CAA (CAG)n CAA CAG] motif (unit III) was reported in siblings. In both pairs, the structure of repeats was identical among them. This may suggest that their affected parents—the father in the case of Nakamura et al. and the mother in the authors’ case—were carriers of identical duplication, and the presence of an additional [CAA (CAG)_16_ CAA CAG] motif did not destabilize the whole region during transmission (Table 2). As in the case of Koide et al. [17] this mutation could also be described based on the model of the repeat expansion via an FEN-1-resistant flap formation [7]. There are two possible ways in which such structures form within the CAA(CAG)_16_CAA CAG unit: a hairpin, backflap, or triplex structure between this motif and the preceding repeat unit.

The possible models of CAG/CAA repeat expansion in the *SCA17*/*TBP* gene discussed above are based on the generally accepted replication models of expansion as the best way of explaining human trinucleotide instability. But as it has also been suggested, the formation of the ssDNA secondary structures and their resolution may involve different processes such as the excision of the secondary structure, repair at double-strand breaks by the recombinational mechanisms (gene conversion repair) or end-joining, mismatch repair, or gap repair [5]. The involvement of a particular mechanism depends on the cell cycle or differentiation phase as well as on the age and the cell type. Interestingly, FEN-1 seems to be involved in all those cellular processes as one of the crucial enzymes maintaining genome stability [8].

## 4. Materials and Methods

The search terms for the literature search in PubMed up to 7 July 2024, were as follows:

[ataxia]; [TATA-binding protein]; [CAG expansion] OR [TBP]; [ataxia]; and [CAG/CAA expansion].

In total, 81 records were found; 2 were removed before screening because they were not in English. Among the 79 records assessed for eligibility, 2 studies were a review, 53 publications were not relevant as they did not contain the full sequence of the TBP abnormal allele and no patient data related to the sequence, and 10 considered only animal or cellular models.

In total, 64 records were excluded from further screening.

In total, 17 studies included in the manuscript contained a detailed human sequence of abnormal TBP alleles, 4 reports were additionally included in the analysis based on references in Zuhlke and Burk, 2007 [33], and 1 was our results [38].

Finally, 19 publications (78 cases) were included in this study; the references positions were [17,18,21,22,23,24,25,26,27,28,29,30,31,32,33,34,35,36,37,38].

In the case of 3 patients diagnosed at the Institute of Psychiatry and Neurology, procedures were performed according to EMQN protocols, including *TBP* PCR, as described by Nakamura et al. 2001 [22]. To establish the sequence of the CAG/CAG repeats region, PCR products were cloned in the pJET1.2 vector using the A/T Cloning system [Fermentas], and inserts were sequenced by the Sanger method using the BigDye Terminatorv.3.1 sequencing standard kit [Applied Biosystem] and pJET1.2 forward and reverse sequencing primers.

## 5. Conclusions

SCA17 is inherited in an autosomal dominant manner, and the offspring of affected individuals are at a 50% risk of inheriting the expanded *TBP* allele. The molecular diagnosis of this disorder is established by the identification of an abnormal CAG/CAA repeat expansion in the TBP gene and determining the number of repeats. However, the age of onset, severity, specific symptoms, and progression of the disease is variable and cannot be precisely predicted by family history or the size of expansion. If it can be generally assumed that a higher number of repetitions correlates with an earlier age of onset (late vs. juvenile form of SCA17), its instability, unlike diseases caused by pure repeat tracts, depends on CAG/CAA configurations. The presence of CAA interruptions breaks up the repetitive sequence into shorter homogenous triplet tracts. This may have a stabilizing influence during DNA replication and could reduce strand slippage. There is no apparent segregation of particular phenotypic traits with CAG/CAA repeat tract structures or repeat lengths; however, it was shown that more complex motif rearrangements are identified in less typical cases.

This is why not only the repeats number but also a detailed analysis of the CAG/CAA repeat structure in expanded alleles should not be performed as it may have a prognostic value for affected families—stability/instability during transmission and possible anticipation of the disease.

## Figures and Tables

**Table 1:** Characterization of the CAA/CAG repeats range and configuration in normal and expanded TBP alleles (previously reported). In the case of the expanded alleles, the number of analyzed cases and transmission is pointed out, as well as the stability during transmission and the possible mechanism of mutation.

CAG/CAA Repeat Structure in TBP Gene	Repeats No.	Analyzed Cases	Stability	Possible Mutation Mechanism	Reference
normal					
(CAG)_3_ (CAA)_3_ (CAG)_nI_ CAA CAG CAA (CAG)_nII_ CAA CAG	29–40	157 unrelated alleles		(CAG)_nI/nII_ polymorphism	[18]
expanded					
(CAG)_3_ (CAA)_3_ (CAG)_9_ CAA CAG CAA (CAG)_9_ (CAA)_3_ (CAG)_9_ CAA CAG CAA (CAG)_19_ CAA CAG	63	single case (de novo paternal transmission)	*-*	partial duplication	[17]
(CAG)_3_ (CAA)_3_ (CAG)_9_ CAA CAG CAA (CAG)_26_ CAA CAG	46	3 gen. (6 family members)	ST	(CAG)n_II_ expansion	[21]
(CAG)_3_ (CAA)_3_ (CAG)_6_ CAA CAG CAA (CAG)_28_ CAA CAG	45	1 gen. (2 sibling)	ST	(CAG)n_II_ expansion	[22]
(CAG)_3_ (CAA)_3_ (CAG)_6_ CAA CAG CAA (CAG)_31_ CAA CAG	48	2 gen. (3 family members)	ST	(CAG)n_II_ expansion	
(CAG)_3_ (CAA)_3_ (CAG)_6_ CAA CAG CAA (CAG)_30_ CAA CAG	47	1 gen. (3 siblings)	ST	(CAG)n_II_ expansion	
(CAG)_3_ (CAA)_3_ (CAG)_9_ CAA CAG CAA (CAG)_16_ CAA CAG CAA (CAG)_16_ CAA CAG	55	1 gen. (2 siblings)	ST	partial duplication	
(CAG)_3_ (CAA)_3_ (CAG)_n_ CAA CAG	53–55	2 gen. (mother and 2 siblings)	NST	partial deletion + exp.	[23]
(CAG)_3_ (CAA)_3_ (CAG)_9_ CAA CAG CAA (CAG)_31_ CAA CAG	51	single case	-	(CAG)n_II_ expansion	
(CAG)_3_ (CAA)_3_ (CAG)_9_ CAA CAG CAA (CAG)_23_ CAA CAG	43	single case	-	(CAG)n_II_ expansion	
(CAG)_3_ (CAA)_3_ (CAG)_9_ CAA CAG CAA (CAG)_30_ CAA CAG	50	single case	-	(CAG)n_II_ expansion	[24]
(CAG)_3_ (CAA)_3_ (CAG)_9_ CAA CAG CAA (CAG)_28_ CAA CAG	48	2 gen. (mother and 6 siblings)	ST	(CAG)n_II_ expansion	
(CAG)_3_ (CAA)_3_ (CAG)_9_ CAA CAG CAA (CAG)_24_ CAA CAG	44	single case	-	(CAG)n_II_ expansion	
(CAG)_3_ (CAA)_3_ (CAG)_11_ CAA CAG CAA (CAG)_24_ CAA CAG	46	2 gen. (father and daughter) *	ST	(CAG)n_II_ expansion	[25]
(CAG)_3_ (CAA)_3_ (CAG)_9_ CAA CAG CAA (CAG)_27_ CAA CAG	47 ^#^	single case	-	(CAG)n_II_ expansion	[26]
(CAG)_3_ (CAA)_3_ (CAG)_6_ CAA CAG CAA (CAG)_31_ CAA CAG	48 ^#^	single case	-	(CAG)n_II_ expansion	
(CAG)_3_ (CAA)_3_ (CAG)_9_ CAA CAG CAA (CAG)_44_ CAA CAG	53	1 gen. (2 siblings)	ST	(CAG)n_II_ expansion	
(CAG)_3_ (CAA)_3_ (CAG)_9_ CAA CAG CAA (CAG)_57_ CAA CAG	53–66	2 gen. (father and son)	NST	(CAG)_nII_ expansion	[27]
(CAG)_3_ (CAA)_3_ (CAG)_8_ CAA CAG CAA (CAG)_26_ CAA CAG	45	1 gen. (2 siblings) **	ST	(CAG)n_II_ expansion	[28]
(CAG)_3_ (CAA)_3_ (CAG)_n_ CAA CAG	53–58	2 gen. (father and son)	NST	partial deletion + exp	[29]
(CAG)_3_ (CAA)_3_ (CAG)_9_ CAA CAG CAA (CAG)_25_ CAA CAG	45	1 gen. (2 siblings) **	-	(CAG)n_II_ expansion	[30]
(CAG)_3_ (CAA)_3_ (CAG)_8_ CAA CAG CAA (CAG)_26_ CAA CAG	45	1 gen. (2 siblings) **	-	(CAG)n_II_ expansion	
(CAG)_3_(CAA)_3_ (CAG)_9_ CAA CAG CAA (CAG)_15_ CAA CAG CAA (CAG)_17_ CAA CAG	55	single case	-	partial duplication + exp	[31]
(CAG)_3_ (CAA)_3_ (CAG)_9_ CAA CAG CAA (CAG)_32_ CAA CAG	52	1 gen. (2 cousins)	ST	partial duplication + exp	[32]
(CAG)_3_ (CAA)_3_ (CAG)_n_ CAA CAG	49–53	2 gen. (father and 2 siblings)	NST	partial deletion + exp	[33]
(CAG)_3_ (CAA)_4_ (CAG)_n_ CAA CAG	53–66	2 gen. (father and daughter)	NST	partial deletion + exp	[34]
(CAG)_3_ (CAA)_4_ (CAG)_n_ CAA CAG	54–55, 61	2 gen. (father and 2 daughters)	NST	partial deletion + exp	
(CAG)_3_ (CAA)_4_ (CAG)_n_ CAA CAG	51–51, 52	2 gen. (father and 2 daughters)	NST	(CAG)n_II_ expansion	
(CAG)_3_ (CAA)_3_ (CAG)_9_ CAA CAG CAA (CAG)_28_ CAA CAG	45	single case	-	(CAG)n_II_ expansion	[35]
(CAG)_3_ (CAA)_3_ (CAG)_n_ CAA CAG	50–55	2 family members	NTS	partial deletion + exp	[11]
(CAG)_3_(CAA)_3_ (CAG)_8_ CAA CAG CAA (CAG)_35_ CAA CAG	54	single case (de novo, paternal transmission)	-	(CAG)_nII_ expansion	[36]
(CAG)_3_ (CAA)_3_ (CAG)_12_ CAA CAA (CAG)_13_ CAA (CAG)_16_ CAA CAG	52	2 gen. (father and proband)	?	probably rearrangement pat. expanded repeats	[37]
(CAG)_3_ (CAA)_3_ (CAG)_9_ CAA CAG CAA (CAG)_23_ CAA CAG	43	single case	-	(CAG)_nII_ expansion	
(CAG)_3_ (CAA)_3_ (CAG)_30_ CAA CAG CAA (CAG)_16_ CAA CAG	57	single case	-	(CAG)_nI+nII_ expansion	
(CAG)_3_ (CAA)_3_ (CAG)_9_ CAA CAG CAA (CAG)_35_ CAA CAG	55	single case	-	(CAG)_nII_ expansion	
(CAG)_3_ (CAA)_3_ (CAG)_9_ CAA CAG CAA (CAG)_31_ CAA CAG	51	single case	-	(CAG)_nII_ expansion	
(CAG)_3_ (CAA)_3_ (CAG)_9_ CAA CAG CAA (CAG)_25_ CAA CAG	45	2 gen. (mother and proband)	-	(CAG)_nII_ expansion	
(CAG)_3_ (CAA)_3_ (CAG)_9_ CAA CAG CAA (CAG)_16_ CAA CAG CAA (CAG)_16_ CAA CAG	55	1 gen. (2 siblings)	ST	partial duplication	[38]
(CAG)_3_ (CAA)_3_ (CAG)_11_ CAA CAG CAA (CAG)_27_ CAA CAG	47	2 gen. (mother and son)	ST	(CAG)n_II_ expansion	
(CAG)_3_ (CAA)_3_ (CAG)_9_ CAA CAA AGG (CAG)_3_ (CAA)_3_(CAG)_9_ CAA CAG CAA (CAG)_18_ CAA CAG	56	single case (de novo paternal transmission)	ST	partial duplication	

ST—stable during transmission. NST—unstable during transmission,—stability unknown (single cases), ?—stability unknown no data of probands’ parents, gen.—generation; *—only daughter’s allele was sequenced, we can assume the stable transmission as in both cases the number of the repeats was exactly the same; **—only one sibling’s allele was sequenced, we can assume the stable transmission as in both cases the number of the repeats was exactly the same; ^#^—patient was homozygous for the expanded allele according to repeat number and structure of the region.

**Table 2:** Comparison of the accepted and newly proposed CAA/CAG repeat motifs’ organization in the *TBP* gene. The possible influence of the motifs’ organization on the mutation mechanism and regional stability during transmission (based on already published cases listed in Table 1).

CAG/CAA Motif Organization in *TBP* Gene	
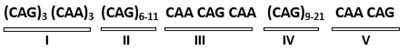	Five-unit organization of the CAG/CAA repeats region according to Gostout et al., 1993 [18]. Two polymorphic (CAG)_n_ tracks are as follows: **II**—less variable *n* = 6–11 (*n* = 9; 91,7%), **IV**—*n* = 9–21 (*n* = 14–17; 83%).
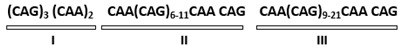	Proposed 3-unit organization of CAG/CAA repeat region. Two motifs of identical structure II and III with variable (CAG)_n_ repeat number. Similar to the five-unit model for a possible mechanism of mutation—expansion of the (CAG)_n_ in unit III as a main mechanism or rearrangements within the region as a result of the interaction (secondary structure formation) between the whole motifs.
Possible model of mutation in identified CAG/CAA motifs	
[(CAG)_3_ (CAA)_2_] [CAA (CAG)_9_CAA CAG] [CAA (CAG)_>28_ CAA CAG]	Expansion of the (CAG)n track in the second CAA(CAG)nCAACAG motif due to its hairpin formation and polymerase slippage—slow, multistep process alleles rather stable in transmission no loss of the basic configuration.
[(CAG)_3_ (CAA)_2_] [CAA (CAG)_45_ CAA CAG]	Expansion of the (CAG)n track as a consequence of its instability after deletion of one of the CAA(CAG)nCAACAG motifs, probably as a result of the hairpin structure formation on the leading strand. Less stable in transmission (in analyzed data max. increased CAG repeats number +7)
[(CAG)_3_ (CAA)_2_] [CAA (CAG)_9_ CAA CAG] [CAA (CAG) _16_ CAA CAG]_2_ [(CAG)_3_ (CAA)_2_] [CAA (CAG)_9_ (CAA)_3_ (CAG)_9_ CAA CAG]_2_ [CAA (CAG)_19_ CAA CAG] [(CAG)_3_ (CAA)_2_] CAA (CAG)_12_ (CAA)_2_ (CAG)_13_ [CAA (CAG)_16_ CAA CAG]	Partial duplication of the CAG/CAA repeats region due to the formation of the FEN1 resistant structures on the 5′ flap end of the Okazaki fragment or by triplex structure forming between the Okazaki fragment and template strand.

## Data Availability

The data presented in this study are available on request from the corresponding author.
